# Web-Based Self-Compassion Training to Improve the Well-Being of Youth With Chronic Medical Conditions: Randomized Controlled Trial

**DOI:** 10.2196/44016

**Published:** 2023-09-13

**Authors:** Amy Louise Finlay-Jones, Asha Parkinson, Fuschia Sirois, Yael Perry, Mark Boyes, Clare S Rees

**Affiliations:** 1 Youth Mental Health Team Telethon Kids Institute Nedlands Australia; 2 School of Population Health Curtin University Bentley Australia; 3 School of Medicine University of Western Australia Crawley Australia; 4 Department of Psychology Durham University Durham United Kingdom

**Keywords:** self-compassion, chronic illness, adolescent psychology, mental health, digital interventions, internet, well-being, mobile phone

## Abstract

**Background:**

Up to one-third of young people live with chronic physical conditions (eg, diabetes, asthma, and autoimmune disease) that frequently involve recurrent pain, fatigue, activity limitations, stigma, and isolation. These issues may be exacerbated as young people transition through adolescence. Accordingly, young people with chronic illness are at a high risk of psychological distress. Accessible, evidence-based interventions for young people with chronic illnesses are urgently needed to improve well-being, support adaptation, and enhance daily functioning. Self-compassion, which is an adaptive means of relating to oneself during times of difficulty, is a promising intervention target for this population.

**Objective:**

This study aims to test the efficacy of a 4-week, self-guided, web-based self-compassion training program for improving well-being among young Australians (aged 16-25 years) living with a chronic medical condition. The primary outcomes were self-compassion, emotion regulation difficulties, and coping; the secondary outcomes were well-being, distress, and quality of life. We also sought to test whether changes in primary outcomes mediated changes in secondary outcomes and gather feedback about the strengths and limitations of the program.

**Methods:**

We conducted a single-blind, parallel-group, randomized controlled trial comparing a 4-week, fully automated, web-based self-compassion training program with a waitlist control. Participants were recruited via the internet, and outcomes were self-assessed at 4 (T1) and 12 weeks (T2) after the baseline time point via a web-based survey. A mixed methods approach was used to evaluate the program feedback.

**Results:**

Overall, 151 patients (age: mean 21.15, SD 2.77 years; female patients: n=132, 87.4%) were randomized to the intervention (n=76, 50.3%) and control (n=75, 49.7%) groups. The loss–to–follow-up rate was 47.4%, and program use statistics indicated that only 29% (22/76) of young people in the experimental group completed 100% of the program. The main reported barrier to completion was a lack of time. As anticipated, treatment effects were observed for self-compassion (*P*=.01; partial η^2^=0.05; small effect); well-being (*P*≤.001; partial η^2^=0.07; medium effect); and distress (*P*=.003; partial η^2^=0.054; small-medium effect) at the posttest time point and maintained at follow-up. Contrary to our hypotheses, no intervention effects were observed for emotion regulation difficulties or maladaptive coping strategies. Improvements in adaptive coping were observed at the posttest time point but were not maintained at follow-up. Self-compassion, but not emotion regulation difficulties or coping, mediated the improvements in well-being.

**Conclusions:**

Minimal-contact, web-based self-compassion training can confer mental health benefits on young people with chronic conditions. This group experiences substantial challenges to participation in mental health supports, and program engagement and retention in this trial were suboptimal. Future work should focus on refining the program content, engagement, and delivery to optimize engagement and treatment outcomes for the target group.

**Trial Registration:**

Australian New Zealand Clinical Trials Registry 12619000572167; https://tinyurl.com/5n6hevt

**International Registered Report Identifier (IRRID):**

RR2-10.1186/s12889-020-8226-7

## Introduction

### Background

Between 13% and 32% of children and young people experience chronic or long-term health conditions, such as cancer, diabetes, autoimmune disorders, asthma, and myalgic encephalomyelitis [1,2]. Compared with their physically healthy peers, these individuals are much more likely to experience mental health difficulties in childhood and adolescence [3], as well as heightened risk of suicidal behavior [4] and mental illness [5] moving into adulthood; for example, a study of individuals aged 6 to 25 years in the United States found a 51% greater adjusted risk of mental health problems among young people with chronic physical conditions compared with those without such conditions [6]. Mental and physical health symptoms can compound one another, leading to a vicious cycle of chronic comorbidities [7]. Consequently, children and young people living with chronic conditions and their sequelae often experience limitations in their participation in school [8] and social activities [9,10] and difficulties in family relationships [11], in addition to the strain of treatment and physical symptoms such as pain and fatigue [12].

Prior reviews have consistently highlighted the need for developmentally appropriate interventions that promote better psychological functioning among youth with chronic illness [13,14]. Accordingly, in early 2018, with support from the Starlight Children’s Foundation, Australia, we commenced a consumer-led research program aimed at promoting well-being and better mental health outcomes among young people with chronic conditions. Across the course of this 3-year program, young people with chronic conditions were consulted on a quarterly basis to discuss their ideas for research, provide feedback on research methods, and guide the interpretation and translation of findings. One priority for these young people was the development of strength-based interventions designed for young people with chronic conditions so that they could access mental health support without needing to rely on a mental health professional. In addition to the difficulties in accessing mental health support, which are frequently reported by young people, having a chronic illness often involves unpredictable and activity-limiting symptoms that can restrict the capacity to access face-to-face services. Accordingly, a strength-based digital intervention is recommended as a feasible means of promoting better well-being and mental health outcomes in this group.

Although there is substantial evidence demonstrating that digital mental health interventions are effective for improving mental health among children and young people [15-22], available digital interventions for young people with chronic conditions are largely limited to disease-specific approaches. Given that young people with different types of chronic conditions share many common experiences and that there are high levels of co-occurring diagnoses in this population, transdiagnostic approaches (ie, those designed for a range of different conditions) have both practical and methodological benefits. However, there are no transdiagnostic digital interventions specifically designed for or in partnership with young people living with chronic conditions. Therefore, we sought to trial a digital program that was tailored to our population of interest.

Self-compassion was considered a reasonable intervention target based on consultations with young people as well as evidence supporting its utility with this target group. As conceptualized by Neff [23,24], self-compassion is an adaptive way of relating to oneself during difficult experiences that involves a tendency toward being compassionate rather than being uncompassionate as a self-response. Compassionate self-responding is characterized by taking a mindful and balanced approach to difficult experiences, recognizing that undergoing such experiences is part of the common human experience, and being kind and understanding toward oneself during times of struggle. In contrast, uncompassionate self-responding is characterized by the tendency to overidentify with difficult experiences, feel alone in one’s difficult times, and treat oneself with judgment or criticism.

Self-compassion is associated with adaptive psychological outcomes in people experiencing both acute [25-27] and chronic stress [28,29]. Among adolescents, meta-analytic evidence demonstrates large inverse effects for the relationship between self-compassion and distress [30], although experimental studies with this population are limited [31]. Previous studies have demonstrated that self-compassion is associated with lower distress and higher quality of life among people living with arthritis [32], cancer [33,34], HIV [35], epilepsy [36,37], and inflammatory bowel disease [38,39]. Importantly, some studies have demonstrated a longitudinal inverse relationship between self-compassion and distress in people with chronic conditions, demonstrating the value of self-compassion as a prospective indicator of mental health problems in this population [39]. Collectively, this evidence suggests that self-compassion has transdiagnostic relevance across groups with different chronic health conditions.

Self-compassion may promote better mental health outcomes for young people with chronic conditions in several ways. However, one of the dominant explanatory models of self-compassion highlights the mechanistic role of emotion regulation [40]. Emotion regulation refers to the capacity to modulate the intensity or duration of emotional experiences [41]. According to Gratz and Roemer [42], adaptive emotion regulation is underpinned by (1) emotional awareness, clarity, and acceptance and (2) the capacity to access emotion regulation strategies, control impulses, and engage in goal-directed behavior when distressed. According to the emotion regulation model of self-compassion, those who are more self-compassionate use more adaptive emotion regulation during times of stress [40,43-45]. In a cross-sectional study of young people with chronic conditions, Prentice et al [45] found support for both a direct path between self-compassion and distress and an indirect path via emotion regulation difficulties. However, only a direct path between self-compassion and well-being was found, suggesting that other factors may mediate the relationship between self-compassion and positive mental health outcomes.

Prior work by Sirois and colleagues [32] suggests an alternative path by which self-compassion may influence mental health outcomes is via improvements in coping. Coping is a related but conceptually distinct construct to emotion regulation that refers to specific emotional or behavioral strategies that individuals may deploy in response to stressful events rather than to emotions more generally. Adaptive coping involves appraising the source of stress in ways that reduce its perceived threat and do not amplify the distress associated with the stressor [46], as well as taking action to address the stressor. As self-compassion fosters both reappraising situations in less harmful ways and not overidentifying with negative states, it follows that self-compassion would promote the use of adaptive coping. Results from a meta-analysis of 133 studies support this proposition by finding that self-compassion is associated with a greater use of adaptive coping strategies [47] and less use of maladaptive strategies. Importantly, evidence suggests that these findings are relevant for coping with the challenges of chronic conditions. Across a combined sample of people with inflammatory bowel disease and those with arthritis, self-compassion was associated with a greater use of adaptive coping strategies (ie, taking actions), less use of maladaptive strategies (ie, disengagement), and, in turn, lower stress [32].

Given the potential benefits of self-compassion–based interventions for young people with chronic conditions, we sought to test Self-Compassion Online, a web-based, self-guided program designed to improve well-being and reduce distress by increasing self-compassion [48]. Because Self-Compassion Online was initially developed for healthy adults, we engaged a co-design group of young people with chronic conditions to help us tailor the program for this target group. The co-design process for the adapted program (named “Uplift” but referred to here as “Self-Compassion Online–Chronic Medical Conditions” [SCO-CMC]) is outlined in our protocol [49] for this study.

### Objective

This study aimed to determine the efficacy of SCO-CMC relative to the waitlist control for improving outcomes in adolescents and young adults living with chronic illness. Self-compassion, emotion regulation, and coping were selected as primary outcomes, and well-being, distress, and quality of life were selected as secondary outcomes. We also aimed to determine whether any improvements in the secondary outcomes were mediated by changes in the primary outcomes.

It was hypothesized that relative to the waitlist control, the self-compassion intervention group would report (1) significant pretest-posttest improvements in self-compassion, coping, and emotion regulation and (2) significant pretest-posttest improvements in psychological distress, quality of life, and well-being. We also hypothesized that changes in all the outcomes would be maintained at the 12-week follow-up and that changes in the secondary outcomes would be mediated by improvements in self-compassion, emotion regulation, and coping. We also sought to assess the strengths and limitations of the program by gathering feedback from participants.

## Methods

### Trial Design and Setting

**Figure 1:**
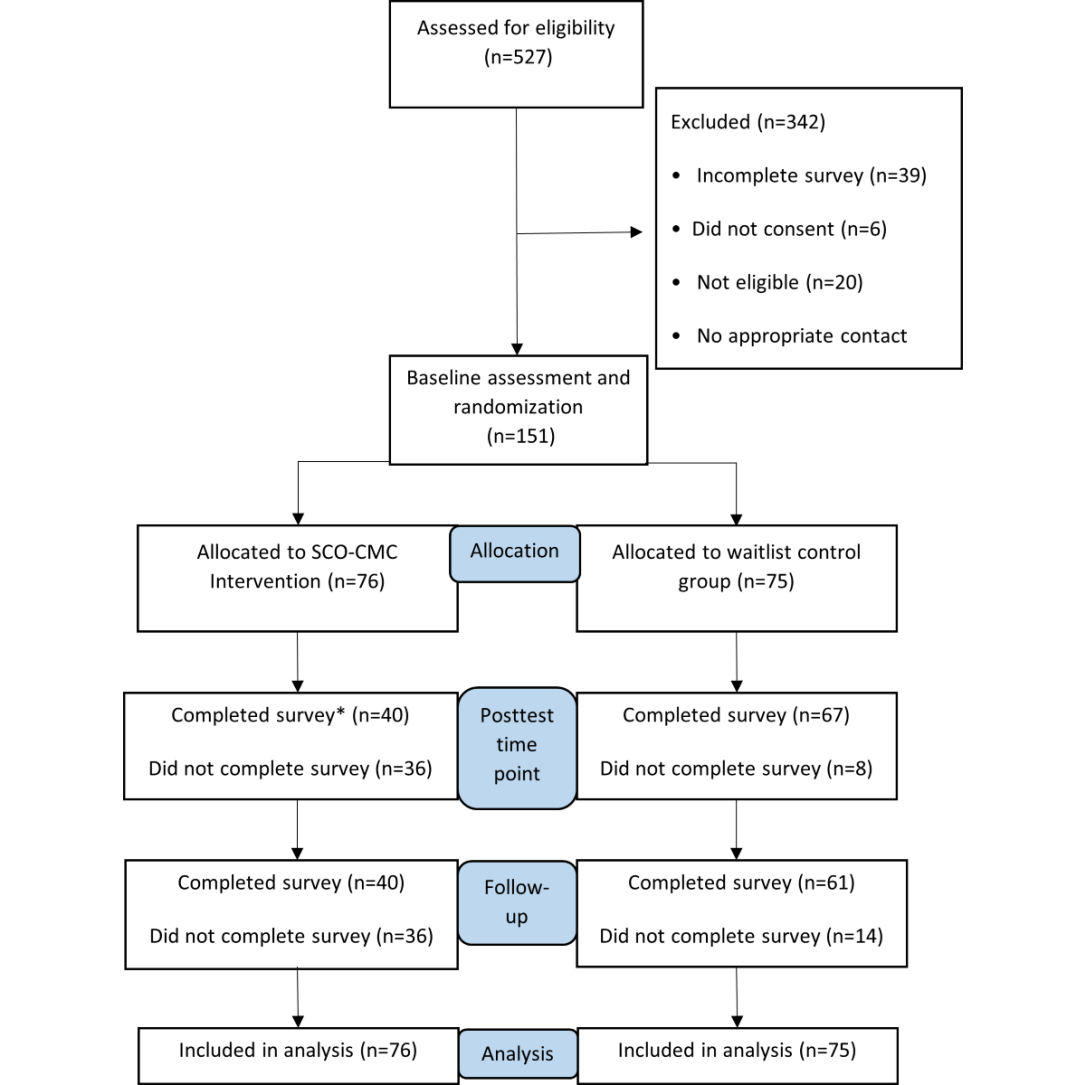
Participant flow through the study. SCO-CMC: Self-Compassion Online–Chronic Medical Conditions. *36 participants provided complete data.

We conducted a parallel-group single-blind randomized controlled trial, comparing the intervention group who had the web-based self-compassion training with the waitlist control. The randomized controlled trial was conducted on the internet, between February 2019 and February 2021. The study design is illustrated in Figure 1.

### Ethics Approval

This study was approved by the Curtin University Human Research Ethics Committee (approval number HRE2019-0386).

### Participants and Recruitment

Participants were recruited on the internet through open-access websites of community organizations that represent various chronic conditions including epilepsy, diabetes, asthma, cystic fibrosis, and myalgic encephalomyelitis or chronic fatigue syndrome. The eligibility criteria were as follows: the participant must (1) be an Australian resident; (2) be aged between 16 and 25 years; (3) be diagnosed as having at least 1 chronic medical condition, which is defined as a physical condition lasting ≥6 months and requiring medical follow-up for >1 year; and (4) have access to a computer and sufficient internet literacy to complete the internet-based program. To facilitate program accessibility, we only required that the participants had a self-reported diagnosis of a chronic medical condition, that is, these were not verified through medical record checks.

### Intervention

**Figure 2:**
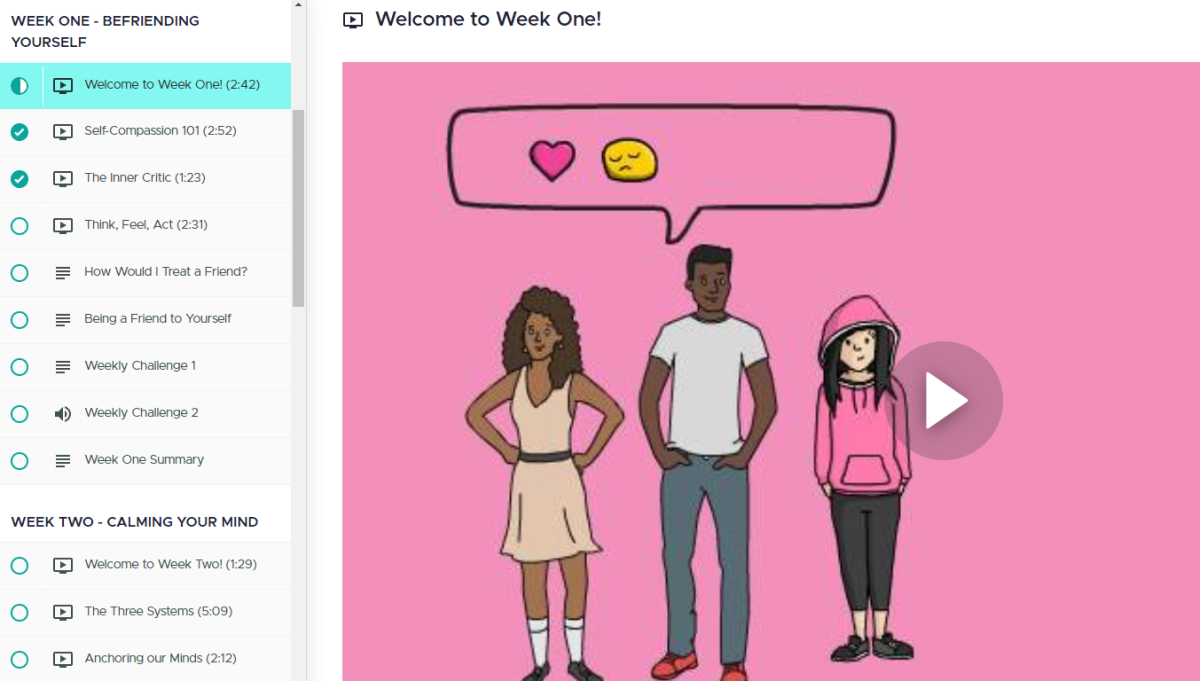
Screenshot of the Self-Compassion Online–Chronic Medical Conditions program.

The intervention was a 4-week fully automated program administered via the internet-based learning management system, Teachable (Teachable, Inc). For the purpose of the trial, once participants were randomized, they were contacted by a research assistant who provided them with log-in details to access the program for free. The program was not publicly available during the study period. The program involved psychoeducational components delivered in text and animated video formats, internet-based forms that participants used for reflection exercises, and meditation exercises delivered in audio and video formats. There was 1 module per week to which the participants were invited to complete at their own pace. All participants received a weekly email reminder to complete the content, regardless of their progress through the program. An overview of the program content was outlined in the protocol [49] for this study. The intervention content did not change during the evaluation period. The research team was not aware of any changes made to the Teachable platform during the evaluation period. A screenshot of the program is shown in Figure 2.

### Outcomes and Measures

#### Overview

All participants were asked to complete the posttest and follow-up measures at 4 and 12 weeks after the baseline, respectively. Upon completion of the follow-up measures, participants in the waitlist group received access to the intervention. All the measures were self-assessed through internet-based surveys administered via Qualtrics (**Qualtrics** International Inc), and participants received a link to the surveys via email. All the measures have previously been validated for internet-based use, and there were no changes in trial outcomes once the trial had commenced. Participants who completed the posttest and follow-up surveys were reimbursed for their time via Aus $10 (US $6.75) vouchers to an Australian retail outlet.

#### Self-Compassion

Self-compassion was measured using the Self-Compassion Scale–Short Form (SCS-SF) [50]. The SCS-SF is a 12-item self-report scale that measures the 6 subcomponents of self-compassion defined by Neff by using a 5-point Likert-type response scale. Subscales that measure uncompassionate self-responding are reverse scored, and the sum of all items is used to yield a total self-compassion score, with higher scores indicating higher levels of self-compassion. The original Self-Compassion Scale and SCS-SF are the most commonly used measures to assess changes in self-compassion via intervention [51].

#### Coping

Coping was measured using the Brief Coping Orientation to Problems Experienced Scale [52], a 28-item scale that measures the use of different coping strategies on a 4-point Likert-type response scale. The Brief Coping Orientation to Problems Experienced Scale consists of 14 subscales. In line with Eisenberg et al [53], we used 12 of these subscales to form 2 composite measures: adaptive coping (comprising active coping, emotional social support, use of instrumental social support, positive reframing, planning, and acceptance subscales) and maladaptive coping (comprising denial, self-distraction, substance use, venting, self-blame, and behavioral disengagement subscales). In this study, adaptive and maladaptive coping strategies were analyzed separately.

#### Emotion Regulation Difficulties

Difficulties in emotion regulation were measured using the Difficulties in Emotion Regulation Scale–Short Form (DERS-SF) [54], an 18-item self-report measure. The DERS-SF measures emotion regulation across six areas: (1) emotional awareness, (2) emotional clarity, (3) emotional acceptance, (4) access to emotion regulation strategies, (5) the ability to engage in goal-directed behavior, and (6) impulse control. In this study, the total emotion regulation difficulty score was calculated by summing the subscale scores. The DERS-SF has demonstrated sound psychometric properties in adolescents and adults [50].

#### Well-Being

Well-being was measured using the World Health Organization Well-Being Index [55], a 5-item self-report measure of subjective well-being. Items are summed and used to calculate a percentage score ranging from 0 to 100. Higher scores indicate higher levels of well-being. The World Health Organization 5-item Well-Being Index has acceptable validity as an outcome measure in intervention trials for both adolescents and adults [55].

#### Distress

Distress was measured using the Kessler Psychological Distress Scale [56]. This 10-item self-report measure examines the frequency of distress over the past 30 days. The item scores are summed to obtain a total distress score. The Kessler 10-item Psychological Distress Scale is sensitive to changes and suitable for use in interventions with young people aged 12 to 25 years [57].

#### Quality of Life

Quality of Life was measured using the Assessment of Quality of Life–Adolescent Version [58]. The Assessment of Quality of Life–Adolescent Version is a 20-item multidimensional measure of health-related quality of life that can be scored as a “psychometric” measure in which the scores of each dimension are unweighted and summed or as a “utility” measure where the dimensions are weighted to facilitate economic evaluation. In this study, the “psychometric” scoring method was used to derive a total score by summing the scores for each of the 6 dimensions. The possible scores range from 20 to 99. The lower the respondent’s score, the better the quality of life.

#### Program Use, Engagement, and Feedback

Data on program engagement and feedback, including barriers to completion, were gathered using 2 mechanisms: via the Teachable platform and via self-report. Data on program log-ins and the completion of individual components of the program were gathered via the Teachable platform. There were two noted issues with these data: (1) participants who did not close their browser or sign out remained logged in and were recorded as a single log-in and (2) for individual component completion, participants had to select “mark as complete” in the Teachable platform for the completion to be recorded. We corroborated these data through self-report: all participants in the intervention group were asked to complete 2 items asking about the proportion of the program they completed and reasons for noncompletion. In addition, interview invitations were sent to a subset of participants in the intervention group 8 weeks after program completion. The interviews focused on participants’ experiences of completing the program, when and how they used the program, what program elements they liked and disliked, and how the program could be improved. Participants were randomly selected at baseline to be invited to an interview to ensure the inclusion of those who may have disengaged from the program. Interviews were conducted with 7 participants with varying levels of program completion.

### Sample Size

An a priori power analysis determined that to conduct a test of intervention effects (power=0.80; Cronbach α=.05) with a medium effect size, 40 participants per group would be required. Due to the complex challenges faced by young people with chronic conditions, we accounted for 45% attrition.

### Randomization

Following recruitment, participants read information sheets on the internet and completed an internet-based consent form by clicking on a check box. Consenting participants were screened, and they completed baseline measures on the internet before randomization, which was a simple randomization conducted using the Qualtrics randomization module.

### Allocation

Once randomized, the participants received a phone call from a research assistant to explain their intervention allocation, including details of how and when they would be able to access the program. This prevented the possibility of multiple enrollments. Participants randomized to the intervention group were enrolled by the research assistant as a Teachable student and received an email link with access to the self-compassion program for 4 weeks.

### Blinding

The researchers who completed the statistical analysis were blinded to the participants’ allocation. Because of the nature of the intervention and the use of the waitlist control, neither the research assistant assisting the implementation of allocation nor the participants were blinded to the intervention allocation.

### Statistical Methods

We conducted intention-to-treat (ITT) analyses of all participants (N=151), and no interim analyses were conducted. In line with the protocol, we also conducted per-protocol analyses, which included all control group participants and all intervention participants who marked >50% of the intervention components as complete. As the per-protocol analysis was conducted after ITT analyses, researchers were no longer blinded to the allocation.

SPSS software (IBM Corp) was used to calculate demographics and mean scores on outcome measures and to compare complete cases with the cases lost, whereas R (version 4.1.1; R Foundation for Statistical Computing) was used for all subsequent analyses. For the hypotheses regarding intervention effectiveness, linear mixed models (LMMs) were used to compare changes in outcome measures across groups and time points while controlling for age and gender. LMMs allow all participants to be retained regardless of attrition and thus are appropriate for ITT analysis [59]. Furthermore, LMMs can account for the lack of independence among an individual’s outcomes measured at different time points and allow the grouping of individuals at higher levels, that is, into intervention and control groups [60].

An LMM was tested for each primary and secondary outcome using the lme4 package for R [61], with a restricted maximum likelihood for missing data. In each model, the participant was included as a random effect, and age was included as a fixed effect. Time (baseline, posttest, and follow-up time points); condition (the intervention vs waitlist control groups); and the interaction between time and condition were included as fixed effects. The time-by-condition interaction was calculated as a test of intervention effects, with partial η^2^ calculated as a between-groups effect size. For models with significant interaction effects, univariate *F* tests were used to determine the main effects of time within the intervention and control conditions and within-group effect sizes over time. Planned contrasts of estimated marginal means were used to determine significant changes in outcomes across specific time points, with Cohen *d* provided as a measure of effect size. To account for multiple comparisons, the Tukey adjustment was used for *P* value calculations.

We also conducted causal mediation analysis in R using the mediation package developed by Tingley et al [62] to test whether self-compassion, difficulties in emotion regulation, adaptive coping, or maladaptive coping would mediate the relationship between intervention effects and the secondary outcomes of well-being, distress, and quality of life. For each model, the significance of the indirect effect was determined using 100,000 simulations and 95% quasi-Bayesian Cis [63].

Descriptive statistics were used to summarize the program engagement data, and descriptive qualitative coding was used for the interview data. The descriptive coding process began with repeated readings of all data extracts. Initial coding was used to determine patterns within the data set, which were discussed by 2 of the authors, both of whom had lived experience of a chronic condition. Subsequent coding was completed independently by 1 of the authors. The coding was conducted in the context of the research question, “What are the experiences of young people completing the SCO-CMC program?” The codes aimed to capture surface-level trends in the data relating to participants’ program experiences and recommendations.

## Results

### Participant Flow

Figure 1 demonstrates the participant flow through the study. A total of 527 entries were recorded for the internet-based screening survey to determine eligibility. The eligible participants (N=151) were randomized to either the intervention (n=76, 50.3%) or the waitlist control group (n=75, 49.7%) after completing the baseline assessment. The participants were contacted to complete the posttest measures 4 weeks after allocation. All the participants were contacted to complete the follow-up measures 12 weeks after allocation and baseline assessment, regardless of whether they completed the posttest measures at 4 weeks. Participants assigned to the waitlist control group received access to the intervention after completing the follow-up measures.

### Missing Data

The final data set included 151 participants, with 76 (50.3%) and 75 (49.7%) participants in the intervention and waitlist control groups, respectively. Of the 76 participants in the intervention group, 39% (n=30) completed both the posttest and follow-up measures, 22% (n=17) provided only 1 of the 2 measures, and 38% (n=29) did not complete either the posttest or follow-up measures. Of the 75 participants in the waitlist control group, 77% (n=58) completed both the posttest and follow-up measures, 16% (n=12) completed only 1 of the 2 measures, and 7% (n=5) did not complete either the posttest or follow-up measures. For each randomization group, a multivariate analysis of variance (MANOVA) was used to assess the differences between complete cases and the cases lost for demographic variables (age and gender) and baseline scores for well-being, quality of life, distress, self-compassion, coping, and difficulties in emotion regulation. For the intervention group, the MANOVA was nonsignificant (*F*_9,65_=0.93; *P*=.57; partial η^2^=0.114), indicating no differences in outcome variables between complete and incomplete cases. For the waitlist control group, the MANOVA was significant (*F*_9,64_=2.54; *P*=.02; partial η^2^=0.263). Analysis of outcome variables indicated that incomplete cases had significantly higher baseline scores for distress (*P*≤.001) and emotion regulation difficulties (*P*=.003).

### Baseline Characteristics

Table 1 reports baseline demographics and clinical characteristics for each group and for the entire sample. Participants (N=151) reported >50 chronic conditions (Multimedia Appendix 1), with the most reported conditions being chronic pain (n=48, 31.8%), type 1 diabetes (n=36, 23.8%), allergies (n=27, 17.9%), asthma (n=27, 17.9%), chronic fatigue syndrome (n=25, 16.6%), inflammatory bowel disease (n=19, 12.6%), postural orthostatic tachycardia syndrome (n=16, 10.6%), and Ehlers-Danlos syndrome (n=16, 10.6%). Participants (73/151, 48.3%) reported being diagnosed with a mental health condition, with depressive disorders (41/151, 27.2%) and generalized anxiety disorder (54/151, 35.8%) being the most reported diagnoses. Mean scores and SDs as well as internal reliabilities and bivariate correlates for all outcome variables at baseline are reported in Tables 1 and 2, respectively.

**Table 1:** Baseline demographics by group.^a^

Characteristics			All participants (N=151)		Intervention group (n=76)		Control group (n=75)
Age (years), mean (SD)			21.15 (2.77)		21.41 (2.68)		20.89 (2.85)
**Gender, n (%)**							
	Female	132 (87.4)		68 (89.5)		64 (85.3)	
	Male	17 (11.3)		7 (9.2)		10 (13.3)	
	Transgender or gender nonconforming	2 (1.3)		1 (1.3)		1 (1.3)	
Mental health condition (any), n (%)			73 (48.3)		36 (47.4)		37 (49.3)
Depressive disorders, n (%)			41 (27.2)		23 (30.3)		18 (24)
Anxiety disorders, n (%)			58 (38.4)		28 (36.8)		30 (40)
Trauma- and stressor-related disorders, n (%)			17 (11.3)		11 (14.5)		6 (8)
Feeding and eating disorders, n (%)			5 (3.3)		5 (6.6)		0 (0)
Obsessive compulsive disorder, n (%)			7 (4.6)		4 (5.3)		3 (4)
Borderline personality disorder, n (%)			2 (1.3)		2 (2.6)		0 (0)
Bipolar disorder, n (%)			2 (1.3)		1 (1.3)		1 (1.3)

^a^At baseline, there were no significant differences between groups in age*,* gender, mental health diagnoses, or outcome measures.

**Table 2:** Correlation analysis of baseline means, internal reliabilities, and coefficients for all outcome variables.

Variables		DERS-SF^a^	Brief COPE-A^b^	Brief COPE-M^c^		WHO-5^d^		K10^e^		AQoL-6D^f^		Values, mean (SD)			Cronbach α		
**SCS-SF^g^**														32.05 (9.73)			.89
	*r*	−0.70	0.33		−0.47		0.47		−0.54		−0.48						
	*P* value	<.001	<.001		<.001		<.001		<.001		<.001						
**DERS-SF**														46.19 (13.39)			.91
	*r*	1	−0.21		0.60		−0.48		0.64		0.54						
	*P* value	—^h^	.01		<.001		<.001		<.001		<.001						
**Brief COPE-A**														31.04 (7.13)			.86
	*r*	−0.21	1		0.11		0.04		−0.01		0.1						
	*P* value	.01	—		.17		.63		.92		.24						
**Brief COPE-M**														23.80 (5.50)			.76
	*r*	0.60	0.11		1		−0.51		0.64		0.56						
	*P* value	<.001	.17		—		<.001		<.001		<.001						
**WHO-5**														44.08 (18.75)			.83
	*r*	−0.48	0.04		−0.51		1		−0.75		−0.76						
	*P* value	<.001	.63		<.001		—		<.001		<.001						
**K10**														25.77 (7.54)			.90
	*r*	0.64	−0.01		0.64		−0.75		1		0.74						
	*P* value	<.001	.92		<.001		<.001		—		<.001						
**AQoL-6D**														48.23 (12.34)			.92
	*r*	0.54	0.1		0.56		−0.76		0.74		1						
	*P* value	<.001	.24		<.001		<.001		<.001		—						

^a^DERS-SF: Difficulties in Emotion Regulation Scale–Short Form.

^b^Brief COPE-A: Brief Coping Orientation to Problems Experienced Scale, Adaptive Subscale.

^c^Brief COPE-M: Brief Coping Orientation to Problems Experienced Scale, Maladaptive Subscale.

^d^WHO-5: World Health Organization 5-item Well-Being Index.

^e^K10: Kessler 10-item Psychological Distress Scale***.***

^f^AQoL-6D: Assessment of Quality of Life–Adolescent Version.

^g^SCS-SF: Self-Compassion Scale–Short Form.

^h^Not applicable.

### Outcomes and Estimation

These results reflect the ITT analyses, which included all participants regardless of missing data or intervention completion. Fixed effects for the primary outcomes are presented in Table 3, and the estimated marginal means for these outcomes are presented in Tables 4 and 5. For self-compassion, there was a significant condition × time interaction, with a small effect size (*F*_2,203.8_=4.72; *P*=.01; partial η^2^=0.05). Specifically, the effect of time was significant in the intervention condition (*F*_2,214.8_=6.26; *P*=.002; partial η^2^=0.06) but not in the control condition (*F*_2,201.1_=0.72; *P*=.49). Pairwise contrasts of estimated marginal means demonstrated that in the intervention group, there was a significant small increase in self-compassion from baseline to the posttest time point (Cohen *d*=0.38; *P*=.02), which was maintained at follow-up (Cohen *d*=0.43; *P*=.005). No significant changes in self-compassion were observed in the control group.

For difficulties in emotion regulation, the interaction between condition and time was nonsignificant at both the posttest and follow-up time points. For adaptive coping, the effect of the condition × time interaction was significant and of medium size (*F*_2,212.2_=6.18; *P*=.002; partial η^2^=0.06). Univariate tests demonstrated a significant small effect of time on adaptive coping in the intervention group (*F*_2,227.6_=4.59; *P*=.01; partial η^2^=0.04) but not in the control group (*F*_2,204.6_=1.80; *P*=.36). In the intervention group, there was a significant increase in adaptive coping scores from baseline to the posttest time point (Cohen *d*=0.30; *P*=.01); however, this was not maintained at follow-up. For maladaptive coping, all fixed effects, including the interaction between time and condition, were nonsignificant. There were no significant changes in maladaptive coping in either the intervention or the control group.

Fixed effects for secondary outcomes are presented in Table 6, with the estimated marginal means for the outcomes reported in Table 7. For well-being, there was a significant medium effect of the interaction between condition and time (*F*_2,212_=7.52; *P*≤.001; partial η^2^=0.07). In the intervention group, there was a significant medium effect of time on well-being scores (*F*_2,220.6_=9.80; *P*≤.001; partial η^2^=0.08). Estimated marginal means demonstrated significant increases in well-being from baseline to both posttest (Cohen *d*=0.50; *P*=.002) and follow-up (Cohen *d*=0.53; *P*<.001). In the control group, the effect of time was nonsignificant (*F*_2,202.6_=0.30; *P*=.75), and no significant changes were detected in well-being across time points.

For distress, there was a significant small-medium interaction effect between condition and time (*F*_2,207.0_=5.95; *P*=.003; partial η^2^=0.054). There was a significant medium effect of time in the intervention group (*F*_2,214.1_=6.71; *P*=.001; partial η^2^=0.06). The estimated marginal means demonstrated that the intervention group had significant small to medium reductions in distress from baseline to the posttest time points (Cohen *d*=0.41; *P*=.009), which was maintained at follow-up (Cohen *d*=0.41; *P*=.005). The effect of time in the control group was nonsignificant (*F*_2,201.0_=0.73; *P*=.48), and no changes in distress were observed in this group. For quality of life, the interaction effect between condition and time was nonsignificant (*F*_2,200.95_=2.72; *P*=.07).

**Table 3:** Fixed effects of time, condition, time × condition, and control variables on self-compassion and emotion regulation.^a^

Predictors	Self-compassion		Emotion regulation	
	Estimated marginal means (95% CI)	*P* value	Estimated marginal means (95% CI)	*P* value
Intercept	32.64 (21.19 to 44.08)	<.001	49.97 (34.27 to 65.67)	<.001
Treatment	0.48 (−2.59 to 3.55)	.76	1.83 (−2.38 to 6.05)	.39
T1^b^	2.76 (0.80 to 4.72)	.006	−1.72 (−4.40 to 0.97)	.21
T2^c^	3.02 (1.13 to 4.90)	.002	−3.58 (−6.16 to −1.01)	.006
Age	−0.22 (−0.56 to 0.49)	.89	−0.22 (−0.94 to 0.50)	.54
Group × T1	1.57 (−5.98 to −1.05)	.005	1.57 (−1.80 to 4.94)	.36
Group × T2	2.43 (−5.36 to −0.47)	.02	2.43 (−0.91 to 5.77)	.15

^a^For condition, the control group was used as the reference point, and for time, baseline was used as the reference point.

^b^T1: posttest time point.

^c^T2: follow-up.

**Table 4:** Fixed effects of time, condition, time × condition, and control variables on coping.^a^

Predictors	Adaptive coping		Maladaptive coping	
	Estimated marginal means (95% CI)	*P* value	Estimated marginal means (95% CI)	*P* value
Intercept	32.31 (24.24 to 40.37)	<.001	26.21 (19.79 to 32.63)	<.001
Treatment	1.06 (−1.21 to 3.33)	.24	0.51 (−1.26 to 2.28)	.57
T1^b^	2.77 (0.90 to 4.64)	.004	−0.97 (−2.29 to 0.36	.15
T2^c^	1.76 (−0.03 to 3.56)	.05	−0.67 (−1.94 to 0.61)	.30
Age	−0.08 (−0.45 to 0.28)	.64	−0.13 (−0.42 to 0.17)	.39
Group × T1	−3.93 (−6.29 to −1.56)	.001	1.05 (−0.63 to 2.72)	.22
Group × T2	−3.04 (−5.38 to −0.70)	.007	1.30 (−0.36 to 2.96)	.12

^a^For condition, the control group was used as the reference point, and for time, baseline was used as the reference point.

^b^T1: posttest time point.

^c^T2: follow-up.

**Table 5:** Estimated marginal means for primary outcomes by intervention condition.

Outcome			Intervention group							Control group					
			Values, mean (SE; 95% CI)		Pairwise comparisons					Values, mean (SE; 95% CI)			Pairwise comparison		
					Mean difference^a^		*P* value					Mean difference			*P* value
**Self-compassion**															
	T0^b^	31.8 (1.10; 29.7-34.0)		N/A^c^		N/A		32.3 (1.11; 30.1-34.5)			N/A			N/A	
	T1^d^	34.6 (1.30; 32.0-37.1)		−2.76		.01		31.6 (1.13; 29.3-33.8)			0.75			.58	
	T2^e^	34.8 (1.26; 32.4-37.3)		−3.02		.005		32.4 (1.15; 30.1-34.7)			−0.10			.99	
**Difficulties in emotion regulation**															
	T0	45.4 (1.51; 42.3-48.2)		N/A		N/A		47.1 (1.52; 44.1-50.1)			N/A			N/A	
	T1	43.5 (1.77; 40.1-47.0)		1.72		.42		46.9 (1.55; 43.9-50.0)			0.15			.99	
	T2	41.7 (1.73; 38.3-45.1)		3.58		.02		45.9 (1.57; 42.8-49.0)			1.15			.54	
**Adaptive coping**															
	T0	30.5 (0.81; 28.9-32.1)		N/A		N/A		31.6 (0.82; 30.0-33.2)			N/A			N/A	
	T1	33.3 (1.04; 31.3-35.3)		−2.77		.01		30.4 (0.84; 28.8-32.1)			1.16			.26	
	T2	32.3 (1.00; 30.3-34.2)		−1.76		.13		30.3 (0.87; 28.6-32.0)			1.28			.22	
**Maladaptive coping**															
	T0	23.5 (0.63; 22.3-24.8)		N/A		N/A		24.0 (0.64; 22.8-25.3)			N/A			N/A	
	T1	22.5 (0.78; 21.0-24.1)		0.97		.33		24.1 (0.65; 22.8-25.4)			−0.08			.99	
	T2	22.8 (0.76; 21.3-24.3)		0.67		.56		24.7 (0.67; 23.3-26.0)			−0.64			.47	

^a^Mean difference calculated by subtracting the posttest time point or follow-up scores from baseline scores.

^b^T0: baseline.

^c^N/A: not applicable.

^d^T1: posttest time point.

^e^T2: follow-up.

**Table 6:** Fixed effects of time, condition, time × condition, and age on secondary outcomes.

Predictors	Well-being			Distress			Quality of life	
	Estimated marginal means (95% CI)	*P* value	Estimated marginal means (95% CI)		*P* value	Estimated marginal means (95% CI)		*P* value
Intercept	39.06 (17.12 to 60.99)	.001	29.78 (20.7 to 38.87)		<.001	50.06 (34.89 to 65.23)		<.001
Condition	3.28 (−2.73 to 9.29)	.28	−0.52 (−2.96 to 1.91)		.67	−0.56 (−4.48 to 3.37)		.78
T2^a^	8.39 (4.18 to 12.59)	<.001	−2.36 (−3.83 to −0.90)		.002	−0.95 (−2.43 to 0.52)		.20
Age	0.16 (−0.84 to 1.17)	.75	−0.18 (−0.60 to 0.24)		.40	0.08 (−0.77 to 0.62)		.83
Condition × T1^b^	−8.42 (−13.95 to −2.90)	.003	3.03 (1.1 to 4.95)		.002	2.28 (0.36 to 4.21)		.02
Condition × T2	−9.75 (−15.22 to −4.28)	.001	2.62 (0.72 to 4.52)		.007	0.91 (−0.99 to 2.82)		.35

^a^T2: follow-up.

^b^T1: posttest time point.

**Table 7:** Estimated marginal means for secondary outcomes by intervention condition.

Outcome		Intervention group				Control group		
		Values, mean (SE; 95% CI)	Pairwise comparisons		Values, mean (SE; 95% CI)		Pairwise comparisons	
			Mean difference^a^	*P* value			Mean difference	*P* value
**Well-being**								
	T0^b^	42.5 (2.15; 38.3-46.7)	N/A^c^	N/A	45.8 (2.16; 41.5-50.0)		N/A	N/A
	T1^d^	50.2 (2.63; 45.1-55.4)	−7.74	.008	45.1 (2.22; 40.7-49.5)		0.69	.93
	T2^e^	50.6 (2.55; 45.9-55.9)	−8.39	<.001	44.4 (2.27; 39.9-48.9)		1.36	.72
**Distress**								
	T0	26.0 (0.87; 24.3-27.7)	N/A	N/A	25.5 (0.88; 23.8-27.2)		N/A	N/A
	T1	23.7 (1.02; 21.7-25.7)	2.32	.009	26.2 (0.89; 24.4-28.0)		−0.71	.46
	T2	23.6 (1.0; 21.7-25.6)	2.36	.005	25.7 (0.91; 24.0-27.5)		−0.26	.91
**Quality of life^f^**								
	T0	48.5 (1.40; 45.7-51.2)	N/A	N/A	47.9 (1.41; 45.1-50.7)		N/A	N/A
	T1	46.9 (1.51; 43.9-49.8)	1.60	.10	48.6 (1.42; 45.8-51.4)		−0.69	.47
	T2	47.5 (1.49; 44.6-50.5)	0.95	.41	47.9 (1.43; 45.0-50.7)		0.04	.10

^a^Mean difference calculated by subtracting the posttest time point or follow-up scores from the baseline scores.

^b^T0: baseline.

^c^N/A: not applicable.

^d^T1: posttest time point.

^e^T2: follow-up.

^f^Higher scores represent worse quality of life outcomes.

### Ancillary Analyses

The per-protocol analyses are reported in Multimedia Appendix 2. The findings largely reflect those of the ITT analyses. For time × condition interactions, we observed a medium effect for self-compassion (*F*_2,165.2_=5.59; *P*=.004; partial η^2^=0.06); a small effect for adaptive coping (*F*_2,164.4_=4.75; *P*=.01; partial η^2^=0.05); a large effect for well-being (*F*_2,16.3_=12.50; *P*≤.001; partial η^2^=0.14); a medium effect for distress (*F*_2,163.6_=6.50; *P*=.002; partial η^2^=0.07); and a medium effect for quality of life (*F*_2,163.8_=4.39; *P*=.01; partial η^2^=0.05). There were no significant effects on emotion regulation (*P*=.40) or maladaptive coping strategies (*P*=.11).

### Mediating Mechanisms

To determine the potential mechanisms of action, mediation analyses tested whether changes in primary outcomes mediated the relationship of the condition × time interaction with each secondary outcome. Each primary outcome was entered as a fixed effect in the previously specified models. The mediation analyses for well-being, distress, and quality of life are presented in Multimedia Appendices 3-5, respectively. Self-compassion emerged as the only significant mediator between the condition x time interaction and secondary outcomes. For well-being, indirect effects via self-compassion accounted for 15.5% of the total effect of condition × time at the posttest time point and 11.2% of the total effect at follow-up. The indirect effect via self-compassion accounted for 17.3% of the total effect of condition × time on distress at the posttest time point; however, the total effect was no longer significant at follow-up. There were no indirect effects of coping (either adaptive or maladaptive) or difficulties in emotion regulation on any of the secondary outcomes at any time point.

### Program Feedback

Program feedback is summarized in Table 8. Of the 76 participants randomized to the intervention group, 40 (53%) provided program feedback. Due to technical problems with the Teachable platform, the percentage of videos viewed was not recorded. Therefore, we used the completion of individual program components to quantify program completion. However, these statistics represent a conservative estimate, as participants were required to manually mark components as completed and could move ahead through the program without doing this. On the basis of the components marked as completed, 25% (19/76) completed the whole program; for those who did not complete the entire program, the majority (16/23, 70%) reported a lack of time as the main barrier to completion. Encouragingly, most participants who provided program feedback reported using strategies outside the program.

**Table 8:** Program completion and barriers to engagement.

Program feedback		Participants, n (%)
**Percentage of program completed^a^ (n=76)**		
	100%	19 (25)
	>50%	6 (8)
	25%-50%	10 (13)
	<25%	6 (8)
	None	35 (46)
**Reasons for noncompletion (n=23)**		
	Lack of time to complete the program	16 (70)
	Illness interfered with ability to complete program	2 (9)
	Did not find program engaging	2 (9)
	Issues accessing the program	1 (4)
	Competing demands, such as school or work	1 (4)
	Forgot to complete program	1 (4)
**Used program reflection spaces (n=34)**		
	Yes	22 (65)
	No	12 (35)
**Used program strategies outside of program (n=34)**		
	Yes	30 (88)
	No	4 (12)

^a^Percentage was calculated using the number of components manually marked as completed. As participants could progress through the intervention without marking components as completed, we expect that this is a conservative estimate of the completion rates.

During the interviews, young people noted that flexibility was the most significant benefit of the digital intervention format. Participants explained that because of their varying energy levels throughout the day, they appreciated the option to complete the program at any time of the day. This allowed them to choose periods of high energy to work through the content, strengthening their engagement:

I generally have way more energy at night than I do in the morning...So, I did these when I did have energy and I was more focused, and I think that made it easier.

Furthermore, the young people appreciated that digital delivery allowed them to complete the intervention content “at my own pace.” Several referenced prior challenges with engaging in more traditional mental health programs, such as seeing counselors, to demonstrate the contrast and accessibility of internet-based programs. One young person spoke of the “ease” of completing the program on the internet, even in the face of unpredictable symptoms:

If it was on a day where I’m in bed the whole day, I can still do that...It’s not like having to get ready, to go out, go to a group, or go to a counsellor and, you know, make the effort to go do it, which often takes a lot out of people.

Another benefit of digital delivery is the provision of information in various formats including video, audio, and text. Young people expressed varying preferences for information formats, with similar numbers of participants preferring videos to text and vice versa. The ability to choose the format of information allowed young people to learn in the ways that were compatible with their condition; for example, a young person who struggled to look at screens for long periods enjoyed being able to listen to the video content “without missing out on anything.” As the pieces of information presented in texts and videos were similar but not identical, young people recommended a number of features to further improve accessibility. Young people who preferred videos requested a “speech-to-text” function for written information that was not displayed in the videos, whereas those who preferred written information required the ability to view scripts for the video content.

Another area of improvement suggested by several young people was extending the computer-based delivery of the program to mobile devices. Young people who recommended mobile delivery felt that this would improve accessibility, particularly if the program was delivered in a stand-alone mobile app as opposed to the mobile web browser:

I work best through apps, so if I was able to access it through an app on my phone that would be easier.

This was considered an ideal format as it allowed young people to complete the program from anywhere, and phone notifications to complete the program were considered more effective than the email reminders used in this program, with one young person concluding “I know I check my phone more than my emails.” Unsurprisingly, young people who felt that an app format would be more suitable were commonly those who expressed a desire to work through the program in “bite-size” pieces, as opposed to longer sittings. No adverse effects of the program were reported.

## Discussion

### Principal Findings

This study investigated the efficacy of a self-guided, internet-based self-compassion training program to improve self-compassion and related mental health outcomes in young people living with chronic conditions. Outcomes of the participants in the self-compassion program were compared with outcomes of the participants in the waitlist control group, with posttest assessments conducted at 4 weeks and follow-up assessment at 12 weeks. Although the overall results demonstrated the benefits of the program on self-compassion and related well-being outcomes, not all outcomes were as expected. Nevertheless, the findings provide insights into the opportunities and challenges afforded by internet-based programs for young people with chronic conditions, and the baseline profiles of the participants underscore the need for targeted interventions designed for this group.

In line with our hypothesis, improvements in self-compassion were demonstrated in the intervention group at the posttest time point and sustained at follow-up. These findings align with prior work demonstrating that self-compassion is a teachable skill [64]. Although there are relatively few studies that have demonstrated efficacy for improving self-compassion in the self-guided internet-based environment [49,65], the findings of this study add to a growing body of literature, demonstrating that self-compassion is a malleable trait that can be cultivated using a variety of different intervention approaches [64] and over relatively brief periods. Furthermore, these findings add to a small body of work exploring the efficacy of self-compassion–based interventions for people living with chronic conditions [66,67], although most prior work has been conducted with adults. Given the broad array of adaptive outcomes associated with self-compassion, including for this target group, the findings of this study are promising, particularly for programs that are low-cost and relatively accessible.

As hypothesized, there were intervention effects for improvements in adaptive coping at the posttest time point; however, these were not maintained at follow-up. Furthermore, no intervention effects were observed for emotion regulation difficulties or maladaptive coping, which are not consistent with emotion regulation theories of self-compassion [40]. This contrasts with previous work demonstrating changes in emotion regulation and coping associated with self-compassion training [68], including prior versions of our internet-based self-compassion program [49]. One reason for this is that prior versions of the program were more explicitly focused on applying self-compassion within an emotion regulation framework. In this version of the program and in line with feedback from our co-design group, the focus of the program was on building positive assets and coping skills (eg, mindfulness, gratitude, and connecting with others) within a self-compassion framework. Accordingly, it is possible that participants had more opportunities to develop adaptive coping skills but fewer opportunities to explicitly address emotion regulation difficulties and maladaptive coping. This raises the question of alternative mechanisms that may underpin the relationship between self-compassion and mental health in this group.

Another plausible reason for the lack of intervention effect on emotion regulation and maladaptive coping is that the duration of this program is too short to change emotion regulation difficulties that are slower to change, particularly when they are more complex or entrenched. Prior research has demonstrated that the impact of intervention dose on emotion regulation outcomes in adolescents is unclear [69]. Understanding the impact of dose is also a priority for future self-compassion intervention research [70]. However, almost half of the participants reported a diagnosed mental health disorder in addition to their chronic physical conditions, and baseline emotion regulation difficulties for our sample were substantially higher than the mean scores reported in previous studies [54,71]. Accordingly, an extended or more explicit focus on addressing emotion regulation difficulties may be warranted for this target group. Furthermore, as advancements in digital technology have enabled greater ecological validity in emotion regulation measurement (eg, the use of ecological momentary assessment via smartphones) [72], it is recommended that future iterations of self-compassion intervention research consider how digital technology can be leveraged to provide state- and context-sensitive measures of emotion regulation. This would allow researchers to more closely map changes in emotion regulation in response to self-compassion practice, as well as determine changes across contexts that are meaningful to young people with chronic conditions (eg, during difficult experiences in health care settings).

As hypothesized, intervention effects were observed for well-being and distress, with improvements maintained at follow-up. Mediation analyses suggested that these changes were driven in part by improvements in self-compassion, although there was no mediating effect of self-compassion on distress at follow-up. Observational studies have consistently documented larger effects for the relationship between self-compassion and distress than for the relationship between self-compassion and well-being [73], whereas prior self-compassion intervention work has largely focused on distress and related outcomes [67]. Given that well-being plays a central role in contemporary models of mental health, such as the dual-continua model [74], the findings of this study regarding the intervention effects on well-being are an important extension to prior work. We recommend that future work use intervention optimization or dismantling designs to determine which components of the internet-based program are associated with treatment gains in these outcomes and whether the inclusion of components that are specifically focused on addressing emotion regulation difficulties improves treatment effects for emotion regulation and distress.

### Practical Implications

The unpredictable nature of chronic conditions underscores the importance of developing programs that are physically and cognitively accessible during periods of physical limitations. As demonstrated in prior research [75], digital delivery is considered an acceptable format for the program, with the flexibility of this method being considered a key strength by young people, particularly given the additional limitations imposed by COVID-19–related social distancing mandates during the intervention period. Furthermore, unlike most programs developed for young people with chronic conditions, this study adopted a transdiagnostic approach, engaging young people with a broad array of chronic conditions. Our findings suggest that such approaches are a feasible and parsimonious means of mental health intervention for young people with chronic conditions, which may be particularly suitable for those who experience multimorbidity [49]. However, further research is required to optimize this program for young people with different accessibility requirements.

Retention in this study was 66.9% (101/151), and Teachable metrics indicated that 46.05% (35/76) of the participants did not complete the program. Although we believe this latter figure to be an overestimate of noncompletion, retention was below the average of 79% reported in a recent systematic review of digital mental health interventions for children and young people (range 15.79%-100%) [76]. One reason for this may be the additional complexities experienced by young people living with chronic physical conditions who simultaneously experience psychological distress. Indeed, our analysis of baseline differences between those who were lost to follow-up and those who were retained suggests that those who were more distressed and who had higher emotion regulation difficulties and distress were less likely to complete follow-up measures. Future research should consider the additional barriers to engagement faced by such participants and work to codevelop strategies to ensure that these participants have equitable opportunities to engage in the intervention.

Most of the young people who reported reasons for noncompletion cited lack of time as the primary reason for noncompletion. Although a relatively small proportion of young people cited health concerns as a reason for noncompletion, young people with chronic health conditions often experience demanding health treatment and maintenance regimens that limit the time available for other activities. Furthermore, our interview data revealed that program use was also influenced by symptom fluctuations. On the basis of these findings, an interesting avenue for future research is to design interventions comprising brief components that young people can engage with separately, or in combination, at times that suit them (eg, a series of single-session interventions rather than a 4-week consecutive program) [76,77]. Initial evidence supports the efficacy of brief interventions in improving adolescent mental health; for example, 2 studies have found that single-session internet-based positive psychology interventions reduce adolescent depressive symptoms at 4 [78] and 9 months [79] compared with time-matched control conditions. Schleider et al [80] argued that brief digital interventions are uniquely scalable and have a high level of acceptability among young people, particularly if they are freely available for use on an as-needed basis.

Additional strategies to increase engagement include personalizing the intervention, including features that allow participants to connect with others, and using SMS text message reminders to engage in the program. Participant feedback indicated that the provision of information in various formats was a key element in enhancing accessibility and promoting engagement with the program. Ultimately, given that similar numbers of young people reported preferences for each information format, researchers who are designing internet-based programs for youth with chronic illnesses should ensure that any information supplied is available in a wide variety of formats, giving the young person the ability to choose how they would like to receive the information. In the future, it is recommended that researchers use digital programs that collect program use data in a more nuanced manner than that provided by the Teachable platform to allow for a deeper understanding of program use and attrition patterns.

### Strengths and Limitations

A key strength of this study was engaging young people to co-design adaptations to the program before the trial; this aligns with the recommendation from a recent systematic review of preventative interventions for children and young people: “co-design processes with children and young people should be recognized and reported as a necessary component” of digital health intervention research [81]. Furthermore, the qualitative feedback received provides useful insights into important elements for the design and implementation of digital mental health programs for young people. As one of the first internet-based well-being programs for young people with a range of different chronic conditions, this study provides an important foundation, with clear guidance for optimizing the program and research design for the future. Nevertheless, there were several limitations to the study, including the use of the waitlist control group and reliance on self-reported data. Although this was appropriate given the early stage of testing of the SCO-CMC program and the minimal-contact nature of the program, the use of the waitlist control group can artificially inflate the estimates of treatment effects [82]. As noted, attrition from the study was higher than the previously reported averages for children and young people, which can introduce attrition bias [22]. Although we conducted ITT analyses to account for this, these attrition rates compromised the generalizability of the findings. Furthermore, as we did not assess socioeconomic status or digital literacy, we are unable to comment on whether these variables may influence intervention outcomes. Finally, although the ecological validity of the study was generally high, the use of a research assistant to enroll participants in the intervention or waitlist conditions does represent a threat to external validity.

### Conclusions

This study provides an important proof of concept demonstrating the effectiveness of an internet-based minimal-contact program for improving well-being outcomes in young people living with chronic conditions. Future research should build on these findings by using optimization designs to determine whether the intervention can also drive meaningful changes in distress in this target group.

## Appendix

Multimedia Appendix 1 List of chronic conditions reported by participants.

Multimedia Appendix 2 Results for per-protocol analyses.

Multimedia Appendix 3 Mediation analyses for well-being.

Multimedia Appendix 4 Mediation analyses for distress.

Multimedia Appendix 5 Mediation analyses for quality of life.

Multimedia Appendix 6 CONSORT-eHEALTH checklist (V 1.6.1).

## Abbreviations

DERS-SF: Difficulties in Emotion Regulation Scale–Short Form
ITT: intention-to-treat
LMM: linear mixed model
MANOVA: multivariate analysis of variance
SCO-CMC: Self-Compassion Online–Chronic Medical Conditions
SCS-SF: Self-Compassion Scale–Short Form

## Data Availability

Deidentified data are available on the internet [83].
