# Supplementary material for: Web-Based Self-Compassion Training to Improve the Well-Being of Youth With Chronic Medical Conditions: Randomized Controlled Trial
Source: J Med Internet Res. 2023 Sep 13;25:e44016. doi: 10.2196/44016 (PMC10534292; doi:10.2196/44016)
Supplement: Multimedia Appendix 1 [file jmir_v25i1e44016_app1.docx]

| **Table S1**. *List of Chronic Conditions Reported by Participants (N = 151)* | | |
| --- | --- | --- |
| Condition | *n* | % |
| Chronic Pain | 48 | 31.8 |
| Type 1 Diabetes | 36 | 23.8 |
| Asthma | 27 | 17.9 |
| Allergies | 27 | 17.9 |
| Chronic Fatigue Syndrome | 25 | 16.6 |
| Inflammatory Bowel Disease | 19 | 12.6 |
| Dysautonomia | 18 | 11.9 |
| Ehlers Danlos Syndrome | 17 | 11.3 |
| Endometriosis | 13 | 8.6 |
| Cystic Fibrosis | 12 | 7.9 |
| Arthritis | 10 | 6.6 |
| Chronic Skin Conditions | 9 | 6.0 |
| Fibromyalgia | 7 | 4.6 |
| Idiopathic Hypersomnia | 7 | 4.6 |
| Gastroparesis | 6 | 4.0 |
| Coeliac Disease | 4 | 2.6 |
| Chiari Malformation | 4 | 2.6 |
| Epilepsy | 3 | 2.0 |
| Narcolepsy | 3 | 2.0 |
| Joint Hypermobility Syndrome | 3 | 2.0 |
| Cystic Fibrosis Related Diabetes | 2 | 1.3 |
| Gastroesophageal reflux disease | 2 | 1.3 |
| Hypothyroidism | 2 | 1.3 |
| Lupus | 2 | 1.3 |
| Migraine | 2 | 1.3 |
| Mixed Connective Tissue Disease | 2 | 1.3 |
| Syringomyelia | 2 | 1.3 |
| Adenomyosis | 1 | 0.7 |
| Bladder Paralysis | 1 | 0.7 |
| Cancer | 1 | 0.7 |
| Chronic intestinal pseudo-obstruction | 1 | 0.7 |
| Collagenous Gastritis | 1 | 0.7 |
| Congenital Heart Disease | 1 | 0.7 |
| Factor V Leiden | 1 | 0.7 |
| Full Digestive Tract Paralysis | 1 | 0.7 |
| Grave’s Disease | 1 | 0.7 |
| Hashimoto’s Disease | 1 | 0.7 |
| Idiopathic Thrombocytopenia Purpura | 1 | 0.7 |
| Inappropriate Sinus Tachycardia | 1 | 0.7 |
| Intestinal Dysmotility | 1 | 0.7 |
| Multiple Sclerosis | 1 | 0.7 |
| Nail-patella Syndrome | 1 | 0.7 |
| Neurofibromatosis Type 1 | 1 | 0.7 |
| Polycystic Ovary Syndrome | 1 | 0.7 |
| Pelvic Congestion Syndrome | 1 | 0.7 |
| Pelvic Floor Dysfunction | 1 | 0.7 |
| Proliferative Diabetic Retinopathy | 1 | 0.7 |
| Reynaud's Disease | 1 | 0.7 |
| Scoliosis | 1 | 0.7 |
| Supraventricular Tachycardia | 1 | 0.7 |
| Type 2 Diabetes | 1 | 0.7 |
| Temporomandibular Joint Dysfunction | 1 | 0.7 |
| Uveitis | 1 | 0.7 |
| Vocal Cord Dysfunction | 1 | 0.7 |
| Other | 1 | 0.7 |

Note. Participants could report multiple conditions.
