# Supplementary material for: Web-Based Self-Compassion Training to Improve the Well-Being of Youth With Chronic Medical Conditions: Randomized Controlled Trial
Source: J Med Internet Res. 2023 Sep 13;25:e44016. doi: 10.2196/44016 (PMC10534292; doi:10.2196/44016)
Supplement: Multimedia Appendix 2 [file jmir_v25i1e44016_app2.docx]

**Supplementary File 2:** Results for Per-Protocol Analyses

### Tests of Intervention Effects

Fixed effects for primary outcomes are presented in Tables S2 and S3 and estimated marginal means for these outcomes are presented in Table S4. For self-compassion, there was a significant condition x time interaction, with a medium effect size, *F* (2, 165.2) = 5.59, *P*= .004, partial η2 = .06. Specifically, the effect of time was significant in the intervention condition, *F* (2, 163.6) = 5.91, *P*= .003, partial η2 =.07, but not in the control condition, *F* (2, 166.1) = 0.77, *P*= .463. Pairwise contrasts of estimated marginal means demonstrated that in the intervention group, there was a significant increase in self-compassion from baseline to post-test (*P* = .007, *d*= 0.48), which was maintained at follow-up (*P* = .013 *d*= 0.61). There were no significant changes in self-compassion detected in the control group.

For difficulties in emotion regulation, the interaction between condition and time was non-significant at both post-test and follow up. For adaptive coping, the interaction effect of condition by time was significant and of small size, *F* (2, 164.4) = 4.75, *P*= .01, partial η2 = .05. Univariate tests demonstrated a significant, small effect of time on adaptive coping for the intervention group, *F* (2, 164.3) = 3.29, *P*= .04, partial η2 = .04, but not for the control group, *F* (2, 168.2) = 1.81, *P*= .167. For the intervention group, there was a significant increase in adaptive coping scores from baseline to post-test (*P*= .030, *d*= 0.40), however this was not maintained at follow-up. For maladaptive coping, all fixed effects, including the interaction between time and condition, were non-significant. There were no significant changes in maladaptive coping detected in either the intervention or the control group.

Fixed effects for secondary outcomes are presented in Table S5, with estimated marginal means for these outcomes reported in Table S6. For wellbeing, there was a significant, large effect of the interaction between condition and time, *F* (2, 165.3) = 12.50, *P*≤ .001, partial η2 = .14. For the intervention group, there was a significant, large effect of time on wellbeing scores, *F* (2, 163.8) = 13.92, *P*≤ .001, partial η2 = .15. Estimated marginal means demonstrated significant, large increases in wellbeing from baseline at both post-test (*P*≤ .000, *d*= 0.61) and follow-up *(P*< .000, *d* = 0.78). For the control group, the effect of time in the control group was non-significant, F (2, 166.8) = 0.35, *P*= .710, and there were no significant changes in wellbeing detected across time points.

For distress, there was a significant, medium interaction effect between condition and time, *F* (2, 163.6) = 6.50, *P*=.002, partial η2 = .07. There was a significant, medium effect of time in the intervention group, *F* (2, 163.5) = 6.26, *P*= .002, partial η2 = .07. Estimated marginal means demonstrated that the intervention group had small but significant reductions in distress from baseline to post-test (*P*= .005, d = 0.49), which was maintained at follow up (*P*= .010, *d*= 0.46). The effect of time in the control group was non-significant, *F* (2, 165.9) = 0.78, *P*= .460, and no changes in distress were demonstrated for this group.

For quality of life, the interaction effect of condition by time was significant, *F*(2, 163.8) = 4.39, *P*= .014, partial η2 = .05. There was a significant, medium effect of time in the intervention group, *F*(2, 163.2) = 4.39, *P*= .009, η2 = .06. . Estimated marginal means demonstrated that the intervention group had small but significant improvements in quality of life (demonstrated by lower scores on the AQoL-6D) from baseline to follow up, *P*=.010, d= 0.46. Changes in quality of life at post-test were non-significant, *P*=.061. There were no significant changes in quality of life for participants in the control group.

| **Table S2.**  *Fixed Effects of Time, Condition, Time*Condition, and Control Variables on Self-Compassion and Emotion Regulation* | | | | | | | | |
| --- | --- | --- | --- | --- | --- | --- | --- | --- |
|  | Self-Compassion | | | | Emotion Regulation | | | |
| *Predictors* | *Est.* | *CI* | | *P* | *Est.* | *CI* | | *P* |
|  |  | *LL* | *UL* |  |  | *LL* | *UL* |  |
| (Intercept) | 43.10 | 28.10 | 58.10 | <**.001** | 36.50 | 15.66 | 57.34 | <**.001** |
| Treatment | −0.41 | −4.94 | 4.12 | .858 | 4.04 | −2.28 | 10.35 | .209 |
| T1 | 4.04 | 1.45 | 6.63 | **.002** | −2.34 | −6.02 | 1.35 | .213 |
| T2 | 3.91 | 1.23 | 6.60 | **.004** | −4.18 | −7.99 | −0.36 | **.032** |
| Age | −0.50 | −1.14 | 0.14 | .128 | 0.32 | −0.57 | 1.21 | .485 |
| Group*T1 | −4.81 | −7.78 | −1.84 | **.002** | 2.20 | −2.03 | 6.42 | .307 |
| Group*T2 | −3.83 | −6.91 | −0.75 | **.015** | 3.01 | −1.37 | 7.39 | .177 |

Note. LL = Lower Limit; UL = Upper Limit; T1 = Post-Test, T2 = Follow-Up. For condition, the control group was used as the reference point. For time, baseline was used as the reference point.

| **Table S3**.  *Fixed Effects of Time, Condition, Time*Condition, and Control Variables on Coping* | | | | | | | | |
| --- | --- | --- | --- | --- | --- | --- | --- | --- |
|  | Adaptive Coping | | | | Maladaptive Coping | | | |
| *Predictors* | *Est.* | *CI* | | *P* | *Est.* | *CI* | | *P* |
|  |  | *LL* | *UL* |  |  | *LL* | *UL* |  |
| (Intercept) | 33.11 | 22.56 | 43.67 | <**.001** | 24.89 | 15.91 | 33.87 | **<.001** |
| Treatment | 1.61 | −1.78 | 4.99 | 350 | 0.77 | −2.04 | 3.57 | .592 |
| T1 | 3.36 | 0.78 | 5.94 | **.011** | −1.38 | −3.31 | 0.55 | .160 |
| T2 | 1.94 | −0.73 | 4.61 | .154 | −1.83 | −3.82 | 0.17 | .072 |
| Age | −0.15 | −0.60 | 0.30 | .513 | −0.08 | −0.46 | 0.31 | .693 |
| Group*T1 | −4.53 | −7.49 | −1.56 | **.003** | 1.45 | −0.76 | 3.66 | .196 |
| Group*T2 | −3.22 | −6.29 | −0.15 | **.040** | 2.46 | 0.17 | 4.75 | **.036** |

Note. LL = Lower Limit; UL = Upper Limit; T1 = Post-Test, T2 = Follow-Up. For condition, the control group was used as the reference point. For time, baseline was used as the reference point.

| **Table S4**. *Estimated Marginal Means for Primary Outcomes, by Intervention Condition* | | | | | | | | | | | | | | | | | | | |  |
| --- | --- | --- | --- | --- | --- | --- | --- | --- | --- | --- | --- | --- | --- | --- | --- | --- | --- | --- | --- | --- |
| Outcome | **Intervention Group** | | | | | | | | | | **Control group** | | | | | | | | |  |
|  | Mean | SE | 95% CI | | | | Pairwise Comparisons | | | | Mean | | SE | | 95% CI | | | Pairwise Comparison | |  |
|  |  |  | LL | UL | | | Mean Diff ^a^ | | *P* | |  | |  | | LL | | UL | Mean Diff | *P* |  |
| **Self-Compassion** | | | | |  | |  | |  | |  | |  | |  | |  |  |  |  |
| T0 | 32.6 | 2.02 | 28.6 | 36.5 | | |  | |  | | 32.1 | | 1.06 | | 30.0 | | 34.3 |  |  |  |
| T1 | 36.6 | 2.00 | 32.6 | 40.6 | | | −4.04 | | **.007** | | 31.4 | | 1.09 | | 29.2 | | 33.5 | 0.77 | .553 |  |
| T2 | 36.5 | 2.03 | 32.5 | 40.5 | | | −3.91 | | **.013** | | 32.2 | | 1.11 | | 30.0 | | 34.4 | −0.09 | .993 |  |
| **Difficulties in Emotion Regulation** | | | | | | | | | | | | |  | |  | |  |  |  |  |
| T0 | 43.2 | 2.81 | 37.7 | 48.8 | | |  | |  | | 47.3 | | 1.48 | | 44.3 | | 50.2 |  |  |  |
| T1 | 40.9 | 2.79 | 35.4 | 46.4 | | | 2.34 | | .426 | | 47.1 | | 1.52 | | 44.1 | | 50.1 | 0.14 | .990 |  |
| T2 | 39.0 | 2.83 | 33.4 | 44.6 | | | 4.18 | | .082 | | 46.1 | | 1.55 | | 43.0 | | 49.1 | 1.16 | .528 |  |
| **Adaptive Coping** | | | | | | |  | |  | |  | |  | |  | |  |  |  |  |
| T0 | 29.9 | 1.51 | 27.0 | 32.9 | | |  | |  | | 31.5 | | 0.80 | | 30.0 | | 33.1 |  |  |  |
| T1 | 33.3 | 1.49 | 30.4 | 36.2 | | | −3.36 | | **.030** | | 30.4 | | 0.82 | | 28.8 | | 32.0 | 1.16 | .255 |  |
| T2 | 31.9 | 1.53 | 28.9 | 34.9 | | | −1.94 | | .327 | | 30.3 | | 0.85 | | 28.6 | | 31.9 | 1.28 | .219 |  |
| **Maladaptive Coping** | | | | | | |  | |  | |  | |  | |  | |  |  |  |  |
| T0 | 23.3 | 1.25 | 20.8 | 25.7 | | |  | |  | | 24.0 | | 0.66 | | 22.7 | | 25.3 |  |  |  |
| T1 | 21.9 | 1.24 | 19.4 | 24.3 | | | 1.38 | | .339 | | 24.1 | | 0.68 | | 22.8 | | 25.4 | −0.08 | .990 |  |
| T2 | 21.4 | 1.26 | 18.9 | 23.9 | | | 1.83 | | .171 | | 24.7 | | 0.70 | | 23.3 | | 26.0 | −0.63 | .513 |  |
| Note. ^a^ Mean difference calculated by subtracting T1/T2 scores from T0 scores. LL = Lower Limit; UL = Upper Limit; T0: Baseline; T1: Post-Test; T2: Follow-Up  **Table S5.** *Fixed Effects of Time, Condition, Time*Condition, and Age on Secondary Outcomes* | | | | | | | | | | | | | | | | | | | | |
|  | **Wellbeing** | | | | | | | **Distress** | | | | | | | | **Quality of Life** | | | | |
| *Predictors* | *Est.* | *CI* | | | | *P* | | *Est.* | | *CI* | | | | *p* | | *Est.* | | *CI* | | *P* |
|  |  | *LL* | *UL* | | |  | |  | | *LL* | | *UL* | |  | |  | | *LL* | *UL* |  |
| (Intercept) | 52.26 | 23.44 | 81.07 | | | **<.001** | | 24.86 | | 12.61 | | 37.11 | | **<.001** | | 46.08 | | 25.82 | 66.34 | **<.001** |
| Condition | 2.09 | −6.78 | 10.95 | | | .643 | | 0.39 | | −3.29 | | 4.07 | | .672 | | −0.84 | | −6.70 | 5.03 | .779 |
| T1 | 11.24 | 5.57 | 16.91 | | | **<.001** | | **-3.25** | | -5.27 | | -1.22 | | **.002** | | −2.36 | | −4.38 | −0.33 | **.023** |
| T2 | 14.98 | 9.11 | 20.85 | | | **<.001** | | −3.15 | | −5.25 | | −1.06 | | **.003** | | −3.17 | | −5.27 | −1.06 | **.003** |
| Age | −0.41 | −1.64 | 0.82 | | | .510 | | .01 | | −0.51 | | 0.54 | | .961 | | 0.13 | | −0.74 | 1.00 | .771 |
| Condition*T1 | −11.96 | −18.46 | −5.46 | | | **<.001** | | 3.96 | | 1.64 | | 6.28 | | **.001** | | 3.04 | | 0.71 | 5.37 | **.011** |
| Condition*T2 | −16.38 | −23.12 | −9.64 | | | **<.001** | | 3.42 | | 1.01 | | 5.83 | | **.002** | | 3.13 | | 0.71 | 5.54 | **.011** |

Note. LL = Lower Limit; UL = Upper Limit

| **Table S6.** *Estimated Marginal Means for Secondary Outcomes, by Intervention Condition* | | | | | | | | | | | | | |
| --- | --- | --- | --- | --- | --- | --- | --- | --- | --- | --- | --- | --- | --- |
| Outcome | Intervention Group | | | | | |  | Control group | | | | | |
|  | Mean | SE | 95% CI | | Pairwise Comparisons | |  | Mean | SE | 95% CI | | Pairwise Comparisons | |
|  |  |  | LL | UL | Mean Diff. ^a^ | *P* |  |  |  | LL | UL | Mean Diff. | *P* |
| **Wellbeing** |  |  |  |  |  |  |  |  |  |  |  |  |  |
| T0 | 43.5 | 3.95 | 35.7 | 51.3 |  |  |  | 45.6 | 2.08 | 41.5 | 49.7 |  |  |
| T1 | 54.8 | 3.91 | 47.0 | 62.5 | −11.24 | **<.000** |  | 44.9 | 2.14 | 40.7 | 49.1 | 0.72 | .897 |
| T2 | 58.5 | 3.98 | 50.6 | 66.4 | −14.98 | **<.000** |  | 44.2 | 2.19 | 39.9 | 48.5 | 1.39 | .686 |
| **Distress** |  |  |  |  |  |  |  |  |  |  |  |  |  |
| T0 | 25.1 | 1.64 | 21.9 | 28.4 |  |  |  | 25.5 | 0.87 | 23.8 | 27.2 |  |  |
| T1 | 21.9 | 1.63 | 18.7 | 25.1 | 3.25 | **.005** |  | 26.2 | 0.88 | 24.5 | 28.0 | −0.72 | .431 |
| T2 | 22.0 | 1.65 | 18.7 | 25.2 | 3.16 | **.010** |  | 25.8 | 0.90 | 24.0 | 27.6 | −.27 | .897 |
| **Quality of Life**  ^b^ | | | | |  |  |  |  |  |  |  |  |  |
| T0 | 48.8 | 2.60 | 43.6 | 54.0 |  |  |  | 48.0 | 1.38 | 45.2 | 50.7 |  |  |
| T1 | 46.5 | 2.60 | 41.3 | 51.9 | 2.36 | .061 |  | 48.7 | 1.39 | 45.9 | 51.4 | −0.69 | 0.466 |
| T2 | 45.6 | 2.61 | 40.5 | 50.8 | 3.17 | **.010** |  | 47.9 | 1.40 | 45.2 | 50.7 | 0.04 | 0.998 |

Note. ^a^ Mean difference calculated by subtracting T1/T2 scores from T0 scores. ^b^ Higher scores represent worse outcomes for Quality of Life. LL = Lower Limit; UL = Upper Limit
