# Supplementary material for: Web-Based Self-Compassion Training to Improve the Well-Being of Youth With Chronic Medical Conditions: Randomized Controlled Trial
Source: J Med Internet Res. 2023 Sep 13;25:e44016. doi: 10.2196/44016 (PMC10534292; doi:10.2196/44016)
Supplement: Multimedia Appendix 3 [file jmir_v25i1e44016_app3.docx]

| **Table S7**. Secondary Outcomes as Mediators of the Relationship Between the Condition-By-Time Interaction and Wellbeing | | | | | | | | |
| --- | --- | --- | --- | --- | --- | --- | --- | --- |
|  | Post-Test | | | | Follow-Up | | | |
| Variable | Estimate | *p* | 95% CI | | Estimate | *p* | 95% CI | |
|  |  |  | LL | UL |  |  | LL | UL |
| Self-Compassion |  |  |  |  |  |  |  |  |
| ACME | −1.18 | **.016** | −2.67 | −0.14 | −0.98 | **.036** | −2.36 | −0.05 |
| ADE | −6.09 | **.030** | −11.49 | −0.58 | −7.28 | **.009** | −12.70 | −1.92 |
| Total Effect | −7.27 | **.009** | −12.70 | −1.75 | −8.26 | **.003** | −13.76 | −2.73 |
| Difficulties in Emotion Regulation |  |  |  |  |  |  |  |  |
| ACME | −0.31 | .387 | −1.27 | 0.36 | −0.48 | .196 | −1.59 | 0.21 |
| ADE | −6.03 | **.032** | −11.45 | −0.58 | −7.34 | **.008** | −12.85 | −1.81 |
| Total Effect | −6.35 | **.026** | −11.90 | −0.84 | −7.82 | **.006** | −13.36 | −2.18 |
| Approach Coping |  |  |  |  |  |  |  |  |
| ACME | −0.18 | .6715 | −1.31 | 0.91 | −0.14 | .745 | −1.07 | 0.71 |
| ADE | −6.06 | **.031** | −11.60 | −0.51 | −7.33 | **.006** | −12.74 | −1.99 |
| Total Effect | −6.24 | **.024** | −11.70 | −0.81 | −7.47 | **.005** | −12.82 | −2.15 |
| Avoidant Coping |  |  |  |  |  |  |  |  |
| ACME | −0.89 | .215 | −2.50 | 0.54 | −1.10 | .118 | −2.75 | 0.28 |
| ADE | −6.09 | **.027** | −11.49 | −0.66 | −7.26 | **.008** | −12.60 | −1.81 |
| Total Effect | −6.98 | **.015** | −12.54 | −1.45 | −8.36 | **.003** | −13.88 | −2.86 |

Note. ACME = Average Causal Mediation Effect, ADE = Average Direct Effect.
