# Supplementary material for: Web-Based Self-Compassion Training to Improve the Well-Being of Youth With Chronic Medical Conditions: Randomized Controlled Trial
Source: J Med Internet Res. 2023 Sep 13;25:e44016. doi: 10.2196/44016 (PMC10534292; doi:10.2196/44016)
Supplement: Multimedia Appendix 4 [file jmir_v25i1e44016_app4.docx]

| **Table S8.** *Secondary Outcomes as Mediators of the Relationship Between the Condition-By-Time Interaction and Distress* | | | | | | | | |
| --- | --- | --- | --- | --- | --- | --- | --- | --- |
|  | Post-Test | | | | Follow-Up | | | |
| Variable | Estimate | *p* | 95% CI | | Estimate | *p* | 95% CI | |
|  |  |  | LL | UL |  |  | LL | UL |
| Self-Compassion |  |  |  |  |  |  |  |  |
| ACME | 0.43 | **.013** | 0.06 | 0.94 | 0.36 | **.028** | 0.03 | 0.864 |
| ADE | 1.93 | **.033** | 0.17 | 3.76 | 1.40 | .130 | −0.39 | 3.217 |
| Total Effect | 2.37 | **.010** | 0.58 | 4.21 | 1.75 | .061 | −0.08 | 3.57 |
| Difficulties in Emotion Regulation |  |  |  |  |  |  |  |  |
| ACME | 0.25 | .360 | −0.27 | 0.84 | 0.38 | .15 | −0.14 | 0.98 |
| ADE | 1.96 | **.035** | 0.18 | 3.76 | 1.38 | .14 | −0.44 | 3.20 |
| Total Effect | 2.21 | **.023** | 0.35 | 4.10 | 1.76 | .07 | −0.17 | 3.69 |
| Approach Coping |  |  |  |  |  |  |  |  |
| ACME | −0.07 | .688 | −0.45 | 0.30 | −0.05 | .72 | −0.37 | 0.23 |
| ADE | 1.95 | **.036** | 0.13 | 3.79 | 1.38 | .13 | −0.40 | 3.14 |
| Total Effect | 1.89 | **.0240** | 0.09 | 3.68 | 1.33 | .14 | −0.44 | 3.09 |
| Avoidant Coping |  |  |  |  |  |  |  |  |
| ACME | 0.45 | .215 | −0.28 | 1.20 | 0.55 | .118 | −0.14 | 1.31 |
| ADE | 1.94 | **.030** | 0.16 | 3.74 | 1.40 | .115 | -0.36 | 3.20 |
| Total Effect | 2.39 | **.016** | 0.47 | 4.32 | 1.95 | **.043** | 0.06 | 3.90 |

Note. ACME = Average Causal Mediation Effect, ADE = Average Direct Effect.
