# Supplementary material for: Web-Based Self-Compassion Training to Improve the Well-Being of Youth With Chronic Medical Conditions: Randomized Controlled Trial
Source: J Med Internet Res. 2023 Sep 13;25:e44016. doi: 10.2196/44016 (PMC10534292; doi:10.2196/44016)
Supplement: Multimedia Appendix 5 [file jmir_v25i1e44016_app5.docx]

| **Table S9**. *Secondary Outcomes as Mediators of the Relationship Between the Condition-By-Time Interaction and Quality of Life* | | | | | | | | |
| --- | --- | --- | --- | --- | --- | --- | --- | --- |
|  | Post-Test | | | | Follow-Up | | | |
| Variable | Estimate | *p* | 95% CI | | Estimate | *p* | 95% CI | |
|  |  |  | LL | UL |  |  | LL | UL |
| Self-Compassion |  |  |  |  |  |  |  |  |
| ACME | 0.45 | **.028** | 0.03 | 1.05 | 0.37 | **.043** | 0.01 | 0.92 |
| ADE | 1.30 | .171 | −0.56 | 3.19 | −0.25 | .786 | −2.11 | 1.60 |
| Total Effect | 1.74 | .068 | −0.14 | 3.64 | 0.12 | .899 | −1.77 | 2.01 |
| Difficulties in Emotion Regulation |  |  |  |  |  |  |  |  |
| ACME | 0.23 | .36 | −0.25 | 0.82 | 0.35 | 0.16 | −0.13 | 0.96 |
| ADE | 1.32 | .16 | −0.55 | 3.18 | −0.27 | 0.78 | −2.15 | 1.62 |
| Total Effect | 1.54 | .12 | −0.40 | 3.50 | 0.08 | 0.93 | −1.88 | 2.07 |
| Approach Coping |  |  |  |  |  |  |  |  |
| ACME | −0.94 | .64 | −0.55 | 0.34 | −0.07 | 0.67 | −0.45 | 0.27 |
| ADE | 1.31 | .18 | −0.57 | 3.20 | −0.27 | 0.77 | −2.11 | 1.56 |
| Total Effect | 1.21 | .20 | −0.65 | 3.08 | −0.34 | 0.71 | −2.18 | 1.48 |
| Avoidant Coping |  |  |  |  |  |  |  |  |
| ACME | 0.36 | .215 | −0.22 | 1.00 | 0.44 | .12 | −0.11 | 1.10 |
| ADE | 1.30 | .176 | −0.55 | 3.16 | −0.25 | .79 | −2.08 | 1.61 |
| Total Effect | −1.66 | .098 | −0.28 | 3.59 | 0.20 | .85 | −1.70 | 2.15 |

Note. ACME = Average Causal Mediation Effect, ADE = Average Direct Effect.
